# An Assessment of Floral Visitor Diversity and Visitation Patterns Associated with *Bauhinia variegata* L. in a Semi-Urban South African Environment

**DOI:** 10.3390/plants15192966

**Published:** 2026-09-29

**Authors:** Usia Matsila, Mokgaetji Georginah Mokganya

**Affiliations:** 1 Biological Sciences Department, University of Venda, Thohoyandou 0950, South Africa; matsila.usia@gmail.com; 2 Science Foundation Department, University of Venda, Private Bag X5050, Thohoyandou 0950, South Africa

**Keywords:** abundance, UNIVEN, floral visitors, floral traits, coevolution

## Abstract

Pollination-related interactions are essential for maintaining plant reproduction and biodiversity in both natural and human-modified landscapes. Understanding floral visitor assemblages associated with urban and peri-urban vegetation can provide valuable insights into ecological processes that support ecosystem functioning. This study investigated floral visitor diversity, visitation frequency, and floral morphological characteristics of *Bauhinia variegata* L. in a semi-urban environment at the University of Venda, South Africa. Field observations conducted during the flowering season recorded a diverse assemblage of floral visitors, including honeybees (*Apis mellifera* and *Apis cerana*), carpenter bees (*Xylocopa* spp.), sunbirds (*Cinnyris talatala*), butterflies (*Papilio polytes*), paper wasps (*Polistes olivaceus*), and bee flies (*Bombomyia discoidea*). Among these visitor groups, honeybees exhibited significantly higher visitation frequencies than other floral visitors (*p* < 0.001). Measurements of floral structures revealed relatively consistent dimensions of petals, stamens, and styles, indicating a stable floral architecture characterized by exposed reproductive organs and accessible floral rewards. The findings demonstrate that *B. variegata* supports a diverse community of floral visitors within a semi-urban landscape and highlights the prominent role of bees in floral visitation patterns. However, because pollen transfer, fruit set, and reproductive success were not directly assessed, conclusions regarding pollination effectiveness cannot be drawn. Future studies should evaluate the behavioural interactions of floral visitors and their contributions to plant reproductive success. These findings contribute baseline information on the floral visitor ecology of *B. variegata* in South Africa and provide a foundation for future investigations of plant–floral visitor interactions in urban and peri-urban ecosystems.

## 1. Introduction

### 1.1. Background Information

Pollination is a fundamental ecological process that supports plant reproduction, biodiversity maintenance, ecosystem stability, and global food security. However, anthropogenic pressures such as habitat loss, climate change, pesticide use, and urbanization have caused widespread declines in pollinators, threatening ecosystem functioning and biodiversity conservation efforts [1,2]. Both urban and peri-urban landscapes are often viewed as unfavourable for biodiversity, but growing evidence suggests that they can support pollinator communities by providing diverse floral resources and refugia [3].

Understanding plant–pollinator interactions in these environments is therefore important for developing strategies that integrate biodiversity conservation with sustainable urban development. Floral morphology plays a critical role in pollinator attraction and efficiency. Traits such as corolla structure, nectar accessibility, and the arrangement of reproductive organs are shaped by pollinator-driven selection and influence plant reproductive success [4,5]. While some plants rely on specialized pollination systems, others possess generalized floral traits that attract multiple visitor species, although certain visitors may still contribute to potential pollination outcomes.

*Bauhinia variegata* L. is a widely distributed ornamental and medicinal tree that provides an ideal model for investigating these interactions. Previous studies have reported that diverse floral visitors are associated with the species, but bees, particularly honeybees, frequently play a dominant role in pollen transfer and reproductive success [6]. This study examines the relationship between floral morphology and floral visitor assemblages of *B. variegata* in a semi-urban South African environment, focusing on floral visitor diversity and visitation patterns. The research contributes to understanding plant–floral visitor dynamics in human-modified landscapes and highlights the importance of conserving urban floral visitor communities that may serve as potential pollinators, thereby strengthening ecosystem resilience and maintaining essential ecosystem services.

### 1.2. Description of Bauhinia variegata L.

**Figure 1:**
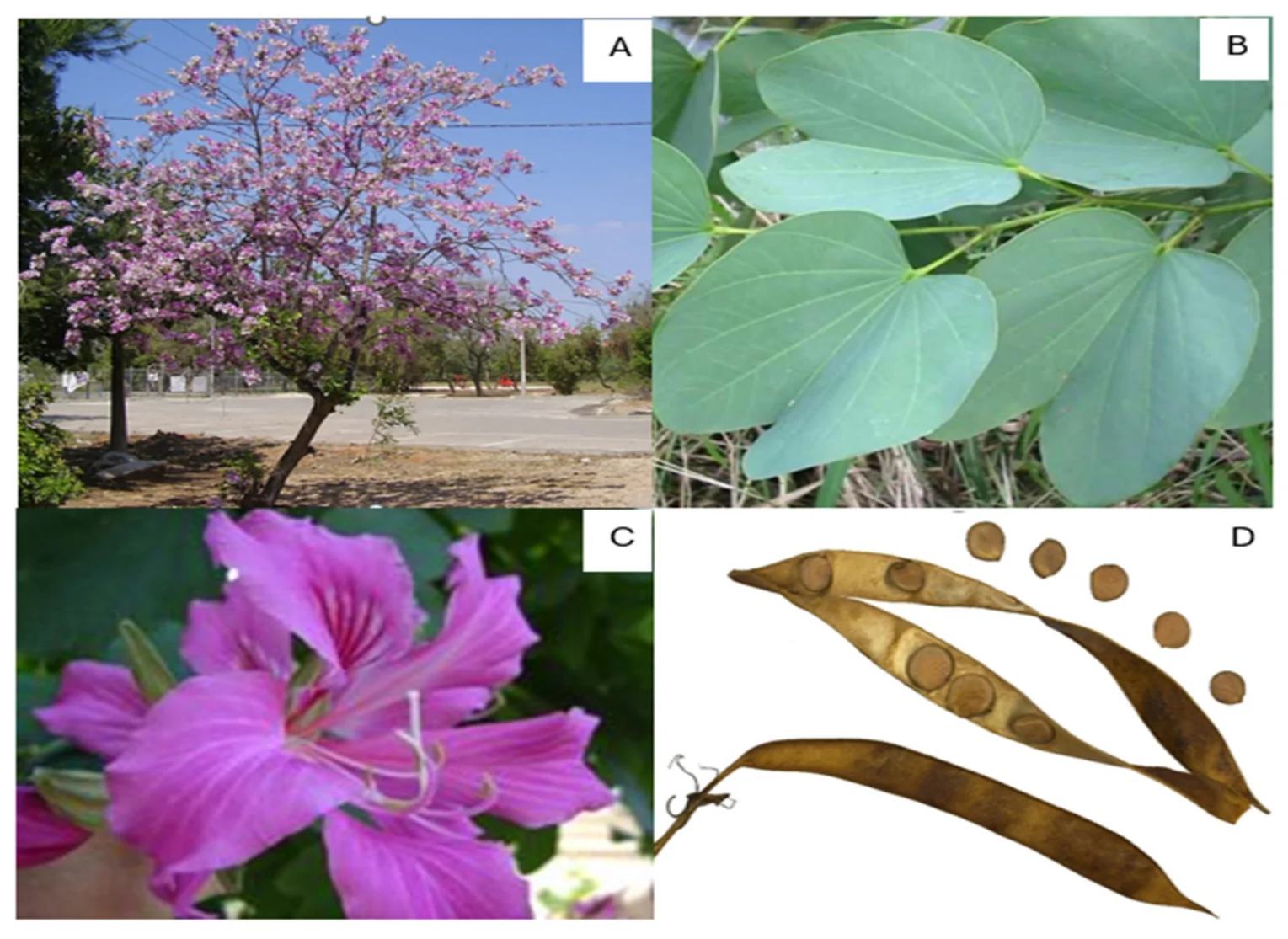
Morphological architecture of *Bauhinia variegata* L. (**A**) is the *Bauhinia variegata* L. tree; (**B**) is the lobed leaves; (**C**) is the floral architectural features, and (**D**) is the pods [11].

*Bauhinia variegata* L., belonging to the botanical family Fabaceae and subfamily Caesalpinioideae, is commonly known as the orchid tree or mountain ebony and is a deciduous, medium-sized tree distributed throughout tropical and subtropical regions [7]. The genus comprises approximately 200–230 species characterized by bilobed leaves (Figure 1B) and hermaphroditic flowers [8]. *Bauhinia variegata* L. typically grows 10–17 m tall (Figure 1A) and produces large, showy, bilaterally symmetrical flowers with five petals, exposed stamens, and an elongated style (Figure 1C), features that enhance pollen accessibility and pollinator efficiency [9,10]. Its open floral architecture and accessible nectar indicate a generalized pollination system that often favours bees. The species also produces elongated seed pods (Figure 1D) and adapts well to diverse soil types while contributing to soil fertility through nitrogen fixation [7].

### 1.3. Phytochemical Attributes of Bauhinia variegata L.

*Bauhinia variegata* L. is rich in bioactive compounds such as flavonoids, tannins, phenolics, saponins, and glycosides, which contribute to its diverse pharmacological properties [12,13]. Studies, such as those of Raj et al. [13], have elucidated that phytochemicals are associated with antioxidant, anti-inflammatory, antimicrobial, antidiabetic, and anticancer activities, highlighting the species’ importance in both traditional and contemporary medicine. Consequently, various plant parts, including the bark, leaves, flowers, buds, and seeds, are widely utilized in Ayurvedic, Unani, and other traditional healthcare systems. Beyond its well-documented medicinal value, *B. variegata* L. also has ecological and socio-economic significance, serving as an ornamental species in urban landscapes and contributing to biodiversity conservation and urban greening initiatives [7]. Additionally, its flowers and buds are consumed as vegetables in several cultures, reflecting their nutritional value.

## 2. Literature Review

### 2.1. Pollination as a Critical Ecosystem Service

Pollination is a vital ecosystem service that supports terrestrial biodiversity, plant reproduction, and food security. Amongst the known pollinators, animal pollinators facilitate the reproduction of approximately 85–90% of flowering plants and contribute substantially to crop productivity and quality. In addition to enhancing plant reproductive success, pollination promotes ecosystem resilience by maintaining genetic diversity and supporting stable ecological networks [14,15]. Furthermore, functional pollinator diversity, rather than species richness alone, plays a key role in ensuring pollination stability and ecosystem resilience [2,16]. However, growing evidence indicates that declines in pollinator diversity and abundance are reducing pollination services and reproductive success, particularly in wild plant communities, highlighting the sensitivity of pollination systems to biodiversity loss and ecological disturbance [16].

### 2.2. Drivers and Dynamics of Pollinator Decline

At present, global pollinator decline is a complex ecological challenge driven by multiple anthropogenic pressures. According to Brunet and Fragoso [1] and Lima et al. [2], nearly 40% of invertebrate pollinator species are estimated to be threatened with extinction. Among the most effective pollinators, bees are particularly vulnerable. Habitat fragmentation and intensive land-use practices reduce the availability of nesting sites and floral resources, weakening pollinator populations and disrupting plant–pollinator interactions. Climate change further exacerbates these challenges by altering flowering and pollinator activity periods, potentially causing phenological mismatches that affect pollination success and ecosystem functioning [1].

Notably, urbanization has traditionally been associated with biodiversity loss. On the other hand, findings from Ayers and Rehan [3] and Remmers and Frentseskaki [17] highlight the potential role of urbanization in pollinator conservation. Furthermore, urban expansion can simplify habitats by well-planning urban green spaces that provide diverse floral resources [3,17]. Ultimately, these heterogeneous habitats can serve as important refugia for pollinators [3,17]. This emerging perspective positions cities as valuable contributors to pollinator conservation and broader sustainability goals.

### 2.3. Floral Morphology, Pollinator Behaviour, and Functional Specialization

Floral morphology serves as the primary indispensable interface between plants and pollinators, influencing pollinator attraction and reproductive success through traits such as corolla structure, nectar accessibility, flower colour, and the placement of reproductive organs [4]. Traditionally, plant species have been classified as specialists or generalists based on their floral characteristics and pollinator assemblages. However, recent studies challenge this distinction by showing that seemingly generalized flowers may exhibit functional specialization. Although many pollinator species may visit a flower, only a limited number often contribute substantially to effective pollen transfer [5,18]. This functional perspective emphasizes the importance of pollinators’ effectiveness rather than focusing on diversity alone. Repeated interactions between specific pollinators and floral traits can drive selection that strengthens trait–pollinator associations over time. Consequently, morphologically generalized species may still display ecological specialization, highlighting the need for integrated approaches that consider floral morphology, pollinator behaviour, and interaction networks when evaluating plant–pollinator relationships [5,18].

### 2.4. Pollination Ecology of Bauhinia variegata L.: Patterns and Knowledge Gaps

The pollination ecology of *Bauhinia variegata* L. demonstrates the interaction between generalized floral morphology and functional specialization. The species produces large, showy flowers with exposed reproductive organs, abundant pollen, and readily accessible nectar resources, characteristics that facilitate visitation by a wide range of floral visitors. Despite this generalized floral architecture, studies consistently indicate that bees, particularly species of *Apis*, are among the most frequent and effective floral visitors because of their ability to efficiently collect and transfer pollen during foraging activities [6,18].

According to Zahid et al. [6], *B. variegata* attracts a diverse assemblage of floral visitors, including bees, flies, butterflies, and other insects; however, bee species account for many floral visits and are likely responsible for most pollen transfer events. Their findings suggest that floral rewards and floral architecture are particularly attractive to bees, which exhibit repeated visitation patterns and high levels of flower constancy. These observations are consistent with broader pollination ecology studies indicating that bee-dominated visitation systems are common among flowering tree species with exposed reproductive structures and accessible nectar resources [19,20]. In parallel, research by Singhal et al. (2005) [18] further demonstrated that *Apis mellifera* and *Apis dorsata* regularly visit *B. variegata* flowers during periods of peak pollen availability, coinciding with anther dehiscence and stigma receptivity. In addition to bees, syrphid flies, beetles, and moths were observed visiting flowers; however, bees were identified as the most effective pollen vectors because of their frequent contact with floral reproductive organs. Likewise, Wani and Chauhan [21] (2008) reported that the synchronization of flower opening, stigma receptivity, and pollen release enhances opportunities for successful pollen transfer by daytime floral visitors.

The importance of floral morphology in determining floral visitor assemblages is further supported by studies across the genus *Bauhinia*. Hokche and Ramírez [22] found that variation in floral size, colour, nectar production, and flower architecture influenced the composition of floral visitor communities, resulting in associations with bees, wasps, butterflies, hummingbirds, and bats. Species possessing open, diurnal flowers with exposed reproductive organs were predominantly visited by insects, especially bees, whereas species with nocturnal flowering traits were associated with bat pollination. These findings suggest that floral architecture serves as an ecological filter, determining visitor accessibility and the utilization of floral resources.

The floral traits of *B. variegata* appear particularly well adapted to bee visitation. Large petals provide strong visual cues, exposed stamens facilitate pollen deposition on visiting insects, and accessible nectar rewards encourage repeated foraging visits. Such traits increase the likelihood of efficient pollen transfer and may explain the dominance of honeybees, which are frequently reported in studies of the species [6,18]. Although *B. variegata* supports a diverse assemblage of floral visitors, visitation diversity does not necessarily translate into equal contributions to pollination. Bees often dominate pollen transport because they possess specialized pollen-collecting structures, exhibit flower constancy, and make numerous visits to rewarding flowers [19,20]. Consequently, *B. variegata* is a useful model for understanding how morphologically generalized flowers can exhibit ecological specialization by relying on a subset of highly effective floral visitors.

Despite increasing research attention, knowledge of *B. variegata* pollination ecology remains geographically biased towards Asia, with relatively limited information available from African ecosystems. Furthermore, most studies have focused on floral biology, breeding systems, and floral visitor diversity, while comparatively few have quantified floral visitation abundance and visitor effectiveness. Additional research examining pollen deposition, pollen transfer efficiency, fruit set, and seed production is therefore required to determine the relative contributions of different floral visitors and to establish whether ecological patterns observed in Asian populations are consistent across African environments [6,18,21].

## 3. Materials and Methods

### 3.1. Study Site Description

**Figure 2:**
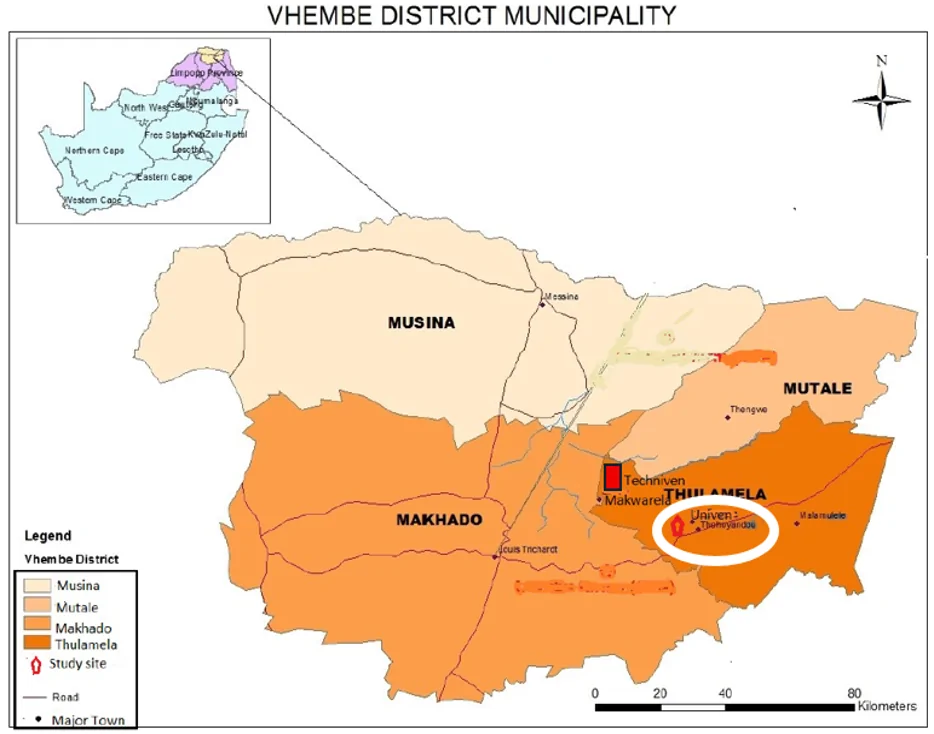
Location of the University of Venda (UNIVEN) within Thulamela Local Municipality, Vhembe District Municipality, Limpopo Province, South Africa. Adopted from [24].

The study was conducted at the University of Venda (UNIVEN), a comprehensive rural-based university located in Thulamela Local Municipality within the Vhembe District Municipality, Limpopo Province, South Africa (Figure 2). The university is situated at approximately 22.9761° S and 30.4465° E, near the town of Thohoyandou, which serves as the administrative and economic center of the Vhembe District. The region is characterized by a mixture of urban, peri-urban, and rural settlements and plays an important role in agricultural production, biodiversity conservation, education, and tourism within Limpopo Province [23,24].

The University of Venda is located within the broader Vhembe landscape, an area recognized for its exceptional biological diversity and ecological significance. The district encompasses portions of the Soutpansberg Mountain Range, protected areas, and biodiversity corridors that contribute substantially to regional conservation efforts. Recent studies have identified the Vhembe District as one of South Africa’s important biodiversity regions, with extensive Critical Biodiversity Areas and Key Biodiversity Areas occurring throughout the municipality [25]. The study area experiences a subtropical climate characterized by warm, wet summers and mild, dry winters. Rainfall is concentrated mainly between October and March, while the winter months receive considerably less precipitation. Climatic variability influences hydrological processes, vegetation dynamics, and agricultural activities throughout the district. Studies conducted within Vhembe District have reported increasing temperatures, greater rainfall variability, and a rising frequency of extreme climatic events, including droughts and floods [26,27]. The Vhembe District has been identified as particularly vulnerable to climate-related hazards due to its reliance on rain-fed agriculture and natural resources. Climate change has altered local livelihood systems, ecosystem functioning, and water resource availability, making environmental monitoring and sustainability research increasingly important in the region [24,26].

The University of Venda (UNIVEN) campus comprises academic facilities, administrative offices, student residences, laboratories, and managed green spaces, providing a suitable environment for environmental and ecological investigations. Furthermore, its location within a rapidly developing rural–urban interface offers opportunities to examine interactions between human activities and environmental systems. The surrounding Vhembe landscape also contains culturally significant ecosystems, including sacred forests that contribute to biodiversity conservation and ecosystem resilience [28].

### 3.2. Sampling Technique

A purposive sampling technique was employed to select the study specimens. Purposive sampling is a non-probability sampling method in which research units are deliberately chosen based on specific characteristics that are relevant to the objectives of the study [29,30]. This technique was considered appropriate because the study specifically focused on *Bauhinia variegata* L., commonly known as the orchid tree, rather than on all tree species occurring within the University of Venda campus. On that note, a total of ten (10) mature *Bauhinia variegata* L. trees were purposively selected from different locations within the University of Venda campus. The selected trees were identified based on species confirmation, accessibility, maturity, structural integrity, and suitability for repeated observations throughout the study period. Only healthy trees exhibiting normal growth characteristics were included in the study, while trees showing visible signs of disease, severe physical damage, dieback, or recent pruning were excluded to minimize potential bias in the observations and measurements. The selected trees were distributed across the study area to capture potential variations in environmental conditions, including sunlight exposure, soil characteristics, human disturbance, and microclimatic influences.

The selection of trees from different locations within the campus was necessitated by the study’s objective, which is obtaining representative observations of *Bauhinia variegata* L. under varying environmental conditions while maintaining the feasibility of repeated field measurements. This choice of ten tree species was necessitated by the intensive nature of field observations and the need to collect detailed information on each sampled individual. Purposive sampling enabled us to focus on information-rich specimens that were most relevant to the study objectives. Essentially, this approach is frequently used in ecological and environmental studies, where species or individuals possess characteristics that are essential to addressing specific research questions [30,31].

### 3.3. Measurements of the Morphological Features

Morphological features such as stem circumference, canopy circumference, sizes of the petals, stamens, and styles were measured. Canopy circumference was determined by first measuring the Canopy diameter in the North–South and East–West directions and averaged. The average of the two measurements was calculated using the equation:

$$\bar{D}=\frac{D_{NS}+D_{EW}}{2}$$

where $\bar{D}$ is the average canopy diameter, $D_{NS}$ is the North–South canopy diameter, and $D_{EW}$ is the East–West canopy diameter [32]. The Canopy circumference was calculated using the formula:

Circumference = π × Diameter

where π ≈ 3.141592653589793.

The stem circumference was measured 30 cm above ground to ensure consistency. The selection of flowers for floral part measurements was based on those found on both sides of the canopy center at distances of 1 m and 2 m. The measuring tape, 30 cm ruler, and Vernier caliper were used to measure the stem diameter, canopy circumference, and floral parts, respectively.

### 3.4. Identification of Flower Visitors of Bauhinia variegata L.

Flower visitors to Bauhinia variegata L. were surveyed to assess diversity and visitation frequency during the flowering period. The study was conducted at two sites on the University of Venda campus: one adjacent to the Finance Building and the other near the Main Gate. Field observations were conducted during the peak flowering season from August to September. Each selected tree was observed on two separate occasions at different times of the day to account for temporal fluctuations in flower-visitor activity. During each observation period, all insects visiting open flowers were recorded, and the number of flower visits was documented to estimate visitation frequency. As alluded to by scholars such as Erickson et al. [33] and O’Connor et al. [34], the direct floral observations are widely recognized as an effective and reliable method for assessing plant–pollinator interactions and quantifying flower visitation rates.

During each observation session, representative flower visitors were collected using a sweeping net to facilitate accurate taxonomic identification. Active sampling methods, such as sweep-net collection, are frequently used in pollinator studies because they capture insects directly associated with floral resources and provide specimens for subsequent laboratory identification [35]. Collected specimens were handled carefully to avoid harm and transported to the laboratory, where they were identified to the lowest possible taxonomic level using morphological characteristics as identification keys. The pollinators were then released after identification. As for the sunbirds, pictures were taken and used for taxonomic identification. The combination of direct observations and sweep-net sampling provided complementary information on both visitation behaviour and flower-visitor diversity. Integrating observational and specimen-based sampling approaches is recommended in pollinator monitoring studies because it improves the accuracy and completeness of floral visitor assessments [34,36].

### 3.5. Monitoring Visitation Rates of Flower Visitors

The visitation rates of flower visitors to *Bauhinia variegata* L. were monitored during the flowering season from August to September. Observations were conducted over a three-week period, with each sampling session lasting three hours per day. To account for potential diurnal variations in flower visitor activity, observations were undertaken during three time intervals: 08:00–11:00, 11:00–14:00, and 14:00–17:00. Monitoring pollinator activity at different times of the day is recommended because visitation patterns are influenced by changes in environmental conditions and floral resource availability throughout the day [33,37]. The abundance of flower visitors was recorded using a handheld tally counter. During each observation period, the number of individual visitors landing on flowers was counted and recorded as an estimate of visitation rate. Direct observation of floral visitors is a widely accepted technique for quantifying pollinator activity and evaluating plant–pollinator interactions because it enables the collection of behavioural and visitation data under natural conditions [33,34].

Furthermore, to minimize the influence of weather-related variability, we ensured that observations were conducted only under favourable environmental conditions. Sampling was therefore restricted to sunny days, and wind speed was maintained below 0.1 m s^−1^, as measured using a Kestrel 4000 pocket weather meter. Weather conditions, particularly wind speed, temperature, and solar radiation, are known to influence pollinator foraging behaviour and visitation frequency; therefore, conducting observations under standardized conditions improves the reliability and comparability of visitation data [33,38].

### 3.6. Data Analysis

#### 3.6.1. Graphical Presentations

The data obtained was presented using bar graphs and heat maps. The choice to use heat maps is justified because they enable simultaneous comparison of multiple variables across all sampled trees. The colour gradient facilitates rapid identification of variations in magnitude and patterns of association, making the data easier to interpret than individual plots.

#### 3.6.2. Statistical Analysis

Data was analyzed using PAST software version 3.1 [39]. The correlation between morphological features was determined using Pearson correlation analysis. On the same note, the statistical difference was determined at *p* < 0.001. Additionally, the mean visitation rates were calculated for each floral visitor group. Differences in visitation rates between groups were evaluated using One-Way Anova. Statistical significance was assessed at *p* < 0.001.

## 4. Results

### 4.1. Measurements of Morphological Features

**Figure 3:**
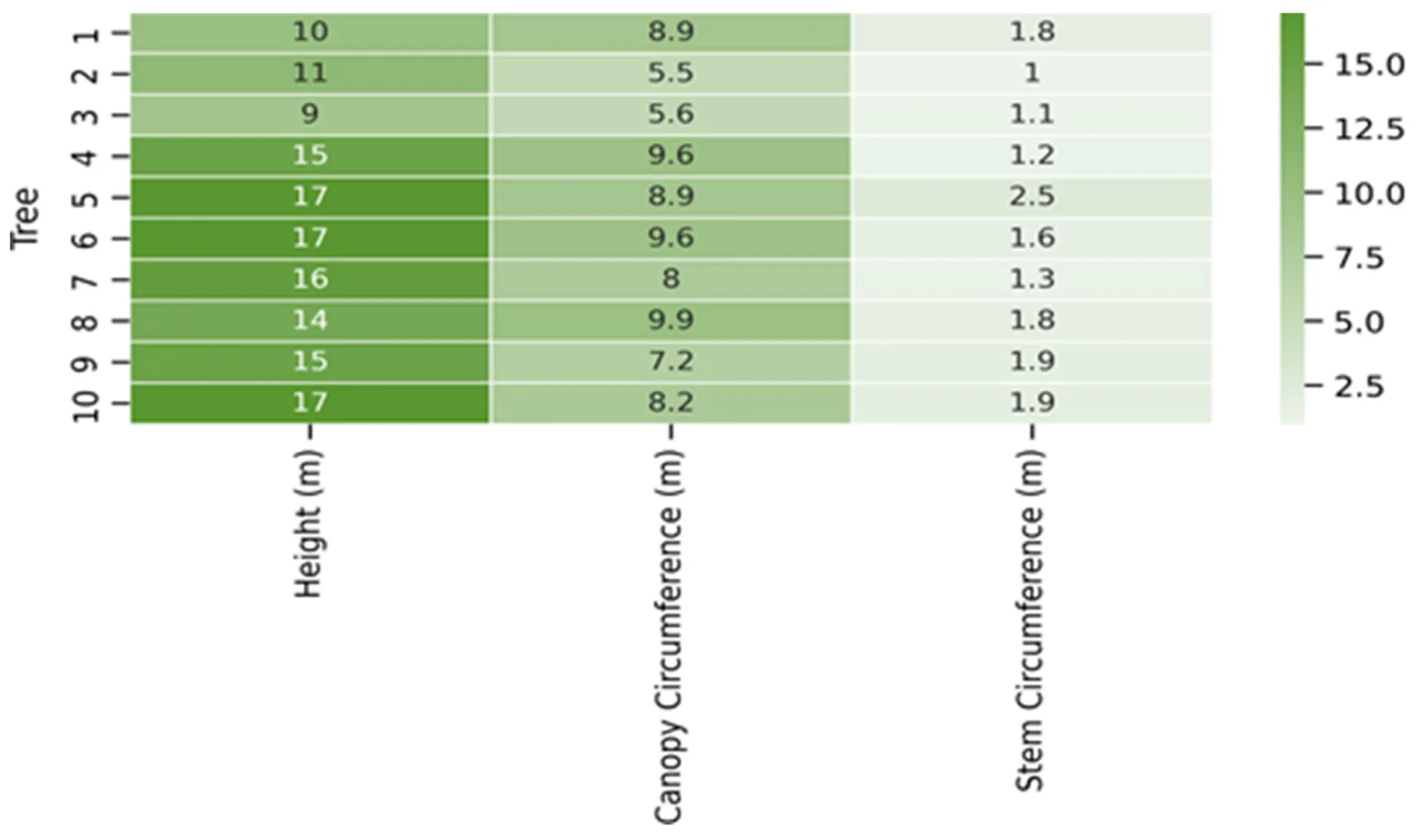
Heat map showing variation in height, canopy circumference, and stem circumference among the sampled *Bauhinia variegata* L. trees. **Note**: the darker colours represent relatively higher values, whereas lighter colours represent lower values.

The morphological attributes measured in the ten sampled trees showed considerable variation (Figure 3), reflecting differences in developmental stage, environmental conditions, resource availability, and competition. Such variability is typical in tree populations because tree architecture is shaped by both intrinsic species characteristics and external ecological factors [40,41].

### 4.2. Tree Height

Tree height ranged from 9 m (Tree 3) to 17 m (Trees 5, 6, and 10). Taller trees generally have greater access to sunlight, which enhances photosynthetic efficiency and biomass accumulation [42,43]. The shorter trees (Trees 1–3) may represent younger individuals or trees experiencing greater competition for resources. Variation in height contributes to stand structural complexity and facilitates resource partitioning within the plant community [40,43].

### 4.3. Canopy Circumference

Canopy circumference varied from 5.5 m to 9.9 m, with Tree 8 exhibiting the largest canopy. Crown size is closely linked to light interception and photosynthetic capacity because larger crowns support greater leaf area and carbon assimilation [40]. Notably, the tallest trees did not have the largest canopies. While Trees 5, 6, and 10 were the tallest, Tree 8 had the widest crown, suggesting that canopy expansion and height growth may follow different allocation strategies. Similar patterns have been reported in previous studies, where crown architecture differed among trees with comparable stem dimensions due to functional traits and ecological adaptations [44]. The large canopy of Tree 8 may therefore reflect an adaptation favouring lateral crown expansion and enhanced light capture [44].

### 4.4. Stem Circumference

Stem circumference ranged from 1.0 m (Tree 2) to 2.5 m (Tree 5). Tree 5 combined the largest stem circumference with one of the greatest heights, indicating advanced structural development and potentially higher biomass accumulation. Stem size is widely recognized as a strong predictor of above-ground biomass and carbon storage because larger stems provide the support required for substantial height and crown growth [43,45]. Conversely, Trees 2 and 3 displayed relatively small stems, shorter heights, and smaller canopies, suggesting lower growth performance or earlier developmental stages. Such relationships between stem dimensions, crown size, and tree height are consistent with established allometric principles [41,46].

### 4.5. Correlations Among Morphological Variables

The heat map (Figure 3) revealed a generally positive relationship among tree height, canopy circumference, and stem circumference. Trees with larger stems typically exhibited greater heights and larger crowns, particularly Trees 5, 6, 8, 9, and 10. This pattern supports allometric theory, which predicts coordinated growth among stem size, crown architecture, and tree height [40,41,46]. However, these relationships were not strictly proportional; for example, Tree 8 had the largest canopy despite not having the largest stem circumference. Such deviations amplify the influence of environmental conditions, species-specific traits, and physiological trade-offs on resource allocation between stem growth and crown expansion [44,46].

**Figure 4:**
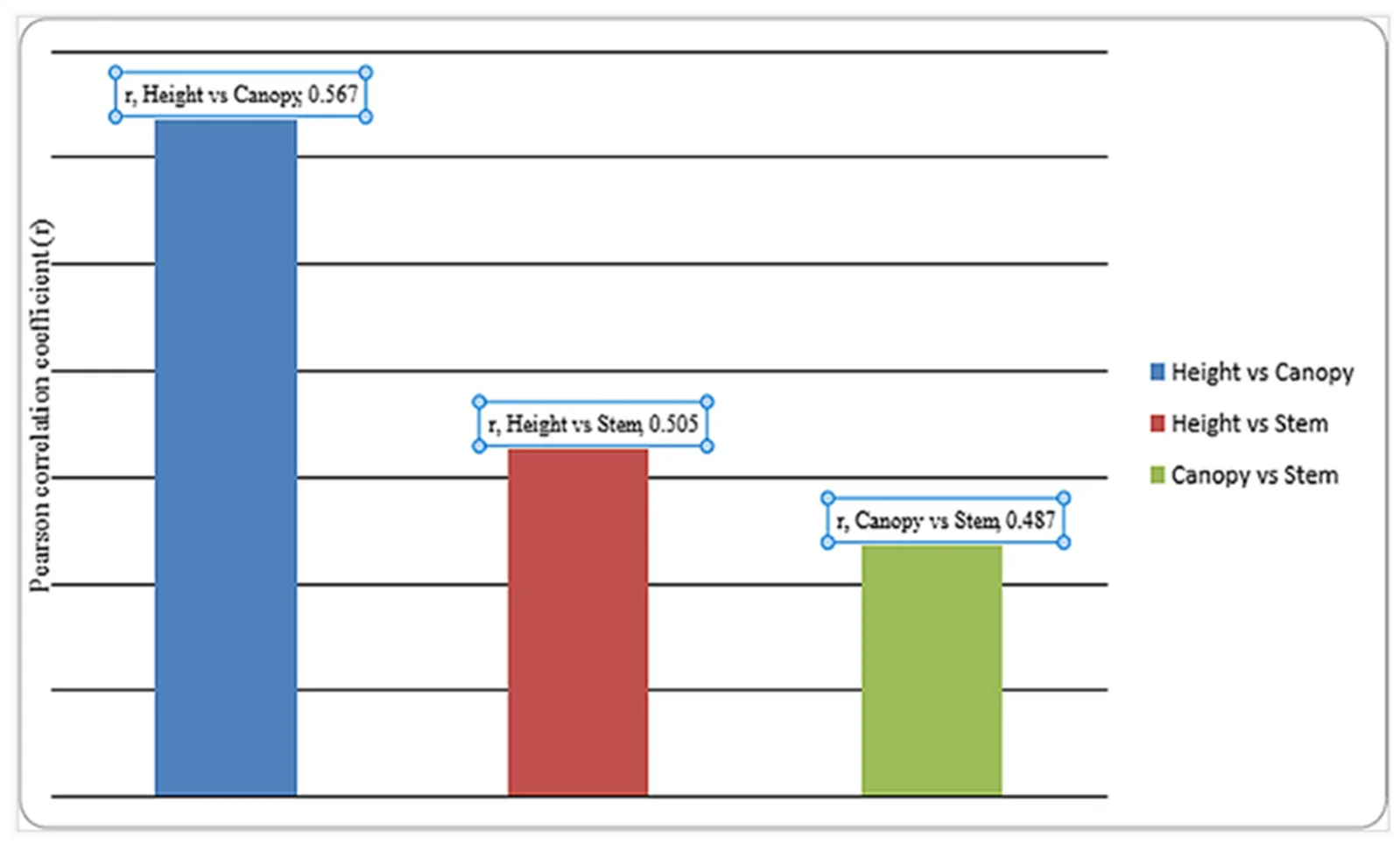
Pearson correlation coefficients among tree height, canopy circumference, and stem circumference. Moderate positive correlations were observed between height and canopy circumference (r = 0.567), height and stem circumference (r = 0.505), and canopy circumference and stem circumference (r = 0.487).

To support this norm, a Pearson correlation analysis was conducted to evaluate relationships among tree height, stem circumference, and canopy circumference, and the results revealed moderate positive relationships among all measured tree attributes (Figure 4). Tree height was positively correlated with canopy circumference (r = 0.567) and stem circumference (r = 0.505), suggesting that taller trees generally exhibited larger canopy spread and thicker stems. Similarly, canopy circumference showed a moderate positive correlation with stem circumference (r = 0.487), indicating that trees with broader canopies tended to possess larger stem girths. These findings suggest that tree growth characteristics are interrelated, with increases in one structural attribute generally associated with increases in others. Even though the analysis highlights positive correlations, none were statistically significant at the 5% significance level.

### 4.6. Architecture of Floral Parts

**Figure 5:**
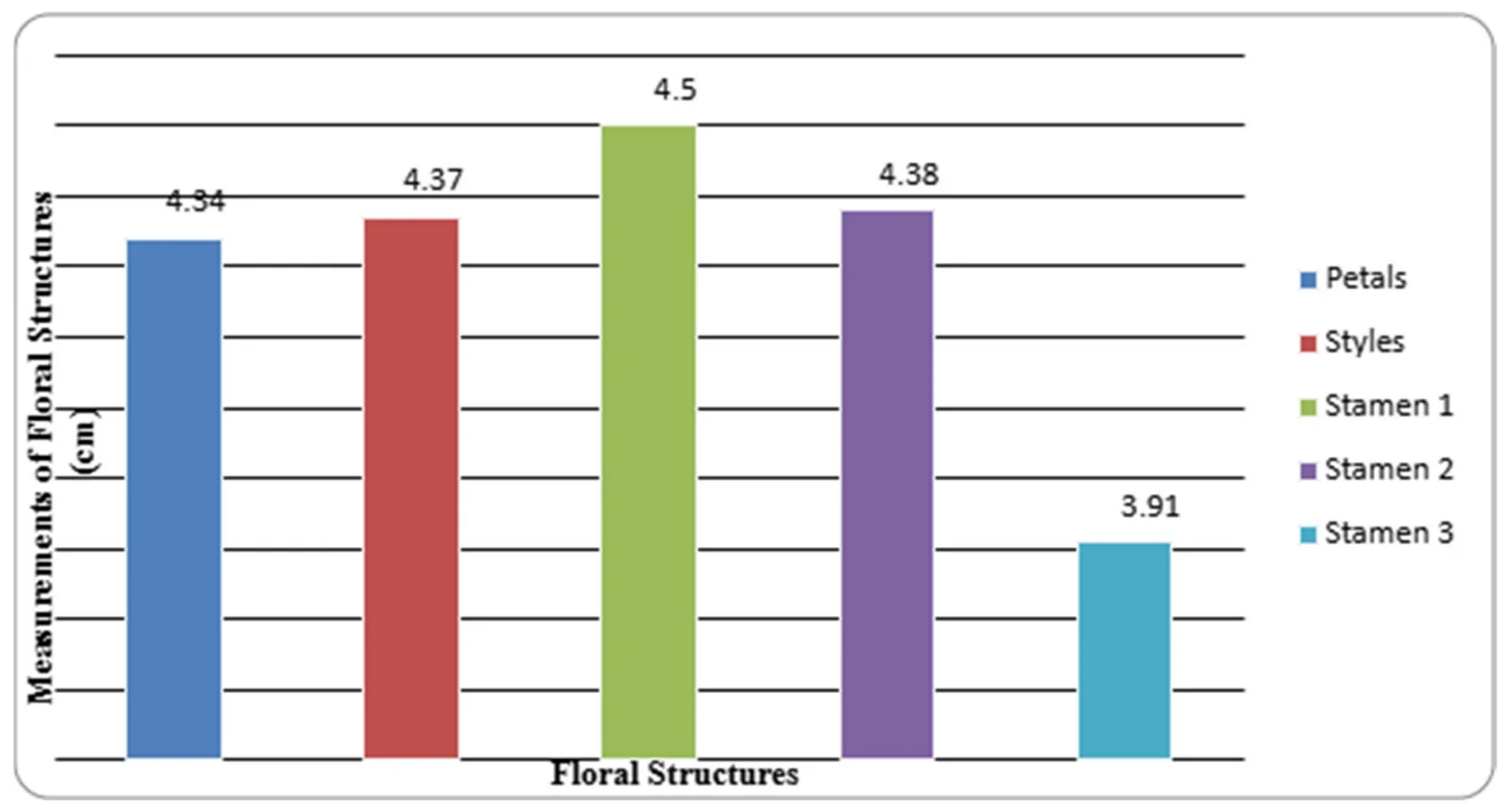
Comparison of the architectural status of the floral parts of *Bauhinia variegata* L.

The floral morphology of *Bauhinia variegata* L. was characterized by relatively stable dimensions across petals, styles, and stamens. Mean petal length was 4.34 cm, mean style length was 4.54 cm, while mean lengths of Stamens 1, 2, and 3 were 4.50 cm, 4.38 cm, and 3.91 cm, respectively (Figure 5). It was also revealed that style length exhibited the least variation among flowers, whereas petal length showed moderate variation. Similarly, the stamens displayed a consistent hierarchical arrangement, with Stamen 1 being the longest and Stamen 3 the shortest. The limited variation observed in style suggests a highly conserved reproductive architectural state. Floral morphometric studies conducted on *Bauhinia* species similarly reported that reproductive structures exhibit relatively consistent dimensions and are closely linked to flowering phenology and reproductive performance [47].

Petal length showed greater variation compared with reproductive structures. Such variability may enhance floral attractiveness by increasing the visual display presented to floral visitors. In *B. variegata*, successful reproduction depends largely on animal-mediated pollination, with floral display traits playing an important role in attracting flower visitors. Findings from recent investigations have demonstrated that the species is visited by diverse insects; however, honeybees, particularly Apis mellifera and Apis dorsata, are the most abundant and anticipated effective pollinators, responsible for pollen transfer and seed production [6].

The observed differentiation among stamen lengths further indicates a coordinated reproductive strategy. Variation in stamen position can improve pollen presentation by increasing the probability that pollen is deposited on different regions of a floral visitor’s body during foraging. This mechanism enhances pollen export efficiency and may increase the likelihood of successful cross-pollination. The maintenance of relatively consistent stamen lengths across flowers suggests developmental stability, facilitating predictable interactions between floral reproductive organs and visiting pollinators [47]. The outcomes further elucidate the close similarity between style length (4.54 cm) and the lengths of the longer stamens (4.50 cm and 4.38 cm), indicating functional coordination between male and female reproductive structures. Such a spatial arrangement promotes effective pollen deposition and retrieval by floral visitors visiting the flowers for nectar or pollen rewards. Corroborating this phenomenon, Zahid et al. [6] reported that bee species achieved the highest levels of pollen deposition and seed set in *B. variegata*, highlighting the importance of floral traits that maximize contact between floral visitors and reproductive organs.

### 4.7. Outcomes of the Identification of Flower Visitors of Bauhinia variegata L.

Table 1 highlights a total of seven taxa belonging to four insect orders and one vertebrate order that were recorded visiting the flowers of *Bauhinia variegata* L. Hymenoptera constituted the dominant group, represented by honeybees (*Apis mellifera* and *Apis cerana*), carpenter bees (*Xylocopa* spp.), and paper wasps (*Polistes* spp.). The predominance of hymenopteran visitors is consistent with previous studies showing that bees are likely to be the principal pollinators of flowering plants due to their high visitation frequency, floral constancy, and ability to transport large quantities of pollen [48,49]. Among the recorded visitors, honeybees (*Apis mellifera* L. and *Apis cerana* Fabricius) and carpenter bees (*Xylocopa* spp.) were observed frequently collecting pollen and nectar and therefore may contribute to pollen transfer. However, pollination effectiveness was not directly measured in the current investigation.

Bees actively forage for pollen and nectar and typically make direct contact with the reproductive structures of flowers, thereby enhancing pollen transfer between flowers and increasing the likelihood of successful fertilization [52,53].

The occurrence of the butterfly *Papilio polytes* indicates that lepidopteran visitors may also contribute to pollination. Butterflies are recognized as supplementary pollinators in many flowering plant communities, particularly for plants with conspicuous and nectar-rich flowers. Although butterflies generally transport less pollen than bees, they may facilitate cross-pollination over longer distances because of their mobility [37,52].

In contrast, the paper wasp (*Polistes* sp.) and the bee fly *Bombomyia discoidea* were categorized as nectar robbers. Nectar robbers often obtain floral rewards without necessarily contacting the reproductive organs of flowers and therefore may contribute less effectively to pollen transfer. Nevertheless, these organisms can still influence plant reproductive success indirectly by altering visitation patterns and competition among effective pollinators [54]. This outlook on the findings suggests that *Bauhinia variegata* L. supports a diverse assemblage of floral visitors, dominated by bee species. This observation is consistent with the general pollination syndrome of many Fabaceae species, which attract a wide range of bee pollinators by producing abundant pollen and nectar [55].

### 4.8. Determination of the Most Frequent Floral Visitors of Bauhinia variegata L.

**Figure 6:**
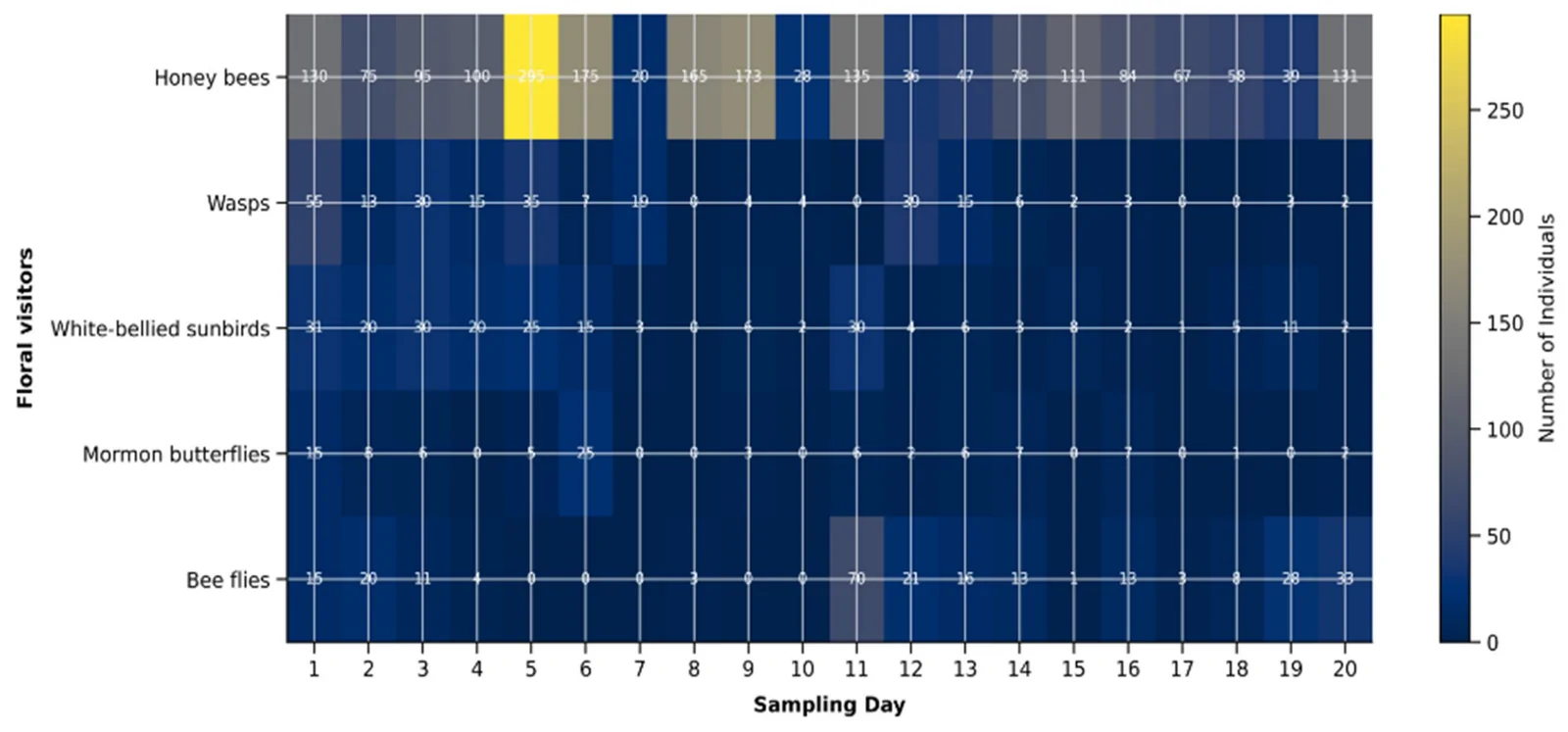
Heatmap illustrating the abundance of floral visitors visiting *Bauhinia variegata* L. across 20 sampling days.

The results clearly indicate that honeybees (*Apis mellifera* and *Apis cerana*) were the most frequent floral visitors observed during the study period. Their visitation rates were substantially higher than those of other flower-visiting taxa, suggesting that honeybees play a dominant role in the pollination ecology of the study species (Figure 6). The high abundance and frequency of honeybee visits may be attributed to their efficient foraging strategies, social organization, and ability to exploit floral resources over large spatial scales [56,57]. Furthermore, differences in visitation frequency among pollinator groups likely reflect variations in resource requirements, foraging behaviour, and temporal activity patterns. The presence of honeybees, birds, and various fly species observed in this study suggests a degree of temporal and ecological niche partitioning, whereby different pollinator groups utilize floral resources at different times or under different environmental conditions, thereby reducing interspecific competition [3,56]. Such resource partitioning promotes the coexistence of multiple pollinator species while enhancing pollination efficiency within plant communities [3,56].

According to Klein et al. [19] and Potts et al. [20], honeybees (*Apis mellifera*) are among the most important pollinators worldwide, contributing substantially to agricultural productivity through crop pollination and supporting the reproduction and persistence of wild flowering plant communities. Animal-mediated pollination is essential for the reproduction of a large proportion of flowering plant species and supports the maintenance of ecosystem biodiversity and food security [58]. Consequently, the predominance of honeybees recorded in this study highlights their ecological importance and their potential contribution to successful pollination and fruit set in the study area.

### 4.9. Statistical Outcomes

**Figure 7:**
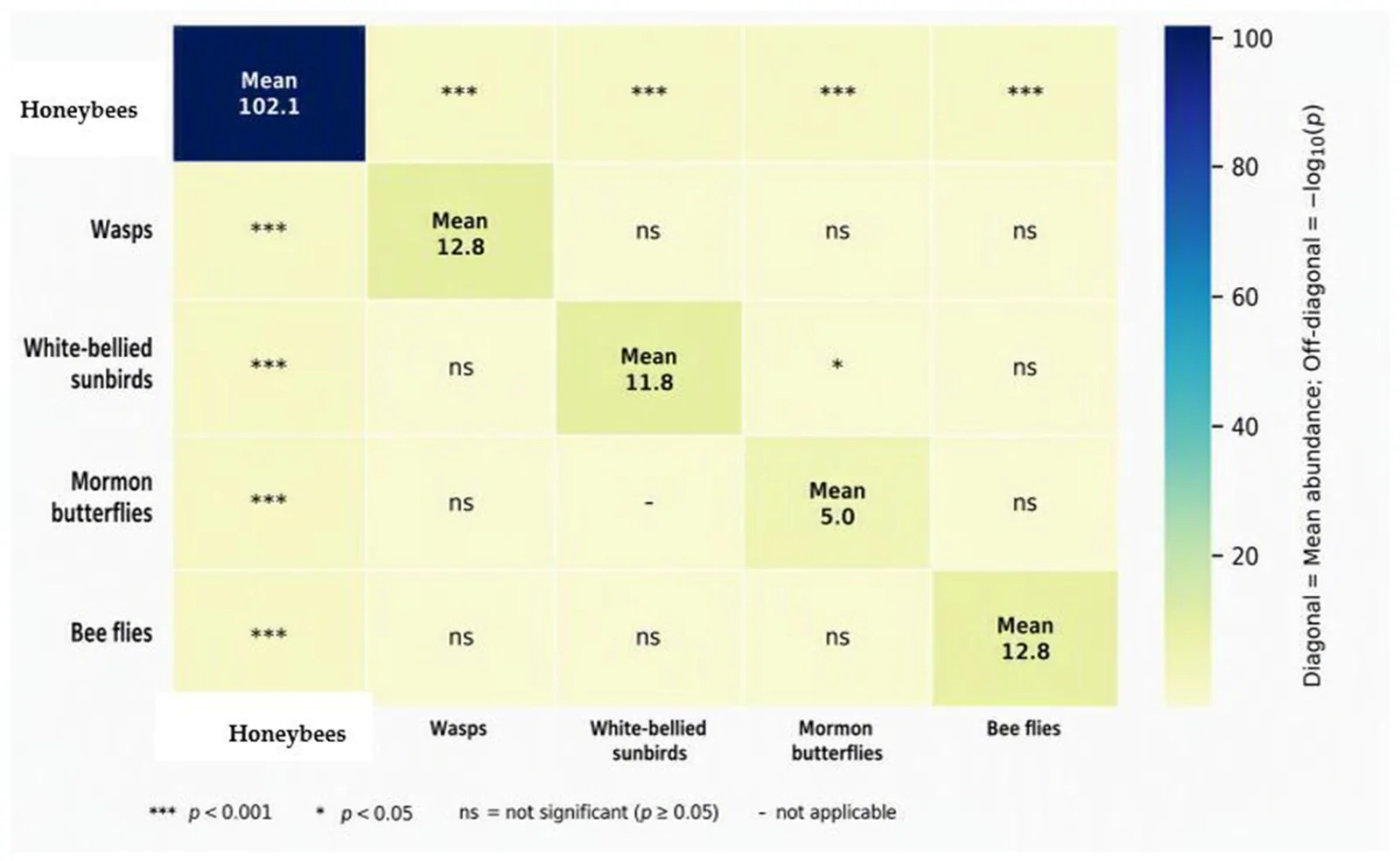
Integrated heatmap illustrating pollinator abundance and statistical significance of pairwise comparisons among pollinator groups visiting *Bauhinia variegata* L. **Note**: Diagonal cells represent the mean abundance (individuals per day^−1^) of each pollinator group, whereas off-diagonal cells represent the significance of pairwise comparisons expressed as −log10(*p*-values). Statistical significance is indicated by asterisks, where *** denotes *p* < 0.001, * denotes *p* < 0.05, and ns indicates non-significant differences.

A one-way comparison of floral visitor abundance revealed remarkable variation among the identified taxa. Honeybees recorded the highest mean abundance (102.1), substantially exceeding the abundance of wasps (12.8), white-bellied sunbirds (11.8), Mormon butterflies (5.0), and bee flies (12.8) (Figure 7). Pairwise comparisons indicated highly significant differences between honeybees and all other floral-visitor groups (*p* < 0.001), demonstrating honeybees’ dominance. No significant differences were observed among wasps, bee flies, and white-bellied sunbirds (*p* ≥ 0.05), although white-bellied sunbirds differed significantly from Mormon butterflies (*p* < 0.05). These findings suggest that honeybees are the most abundant potential pollinator group. The observed dominance of honeybees is consistent with studies reporting that social bees often attain substantially higher population densities than other floral visitors due to large colony sizes and effective foraging strategies [19,20].

From an ecological perspective, floral visitation frequencies of other taxa remain important despite the numerical dominance of honeybees. Different floral visitor taxa vary in their foraging behaviour, floral preferences, and effectiveness, contributing to pollination complementarity and ecosystem resilience [59,60]. Therefore, the relatively lower abundance of wasps, bee flies, butterflies, and white-bellied sunbirds should not be interpreted as indicating a negligible ecological role.

The substantially higher visitation frequency of honeybees suggests a strong functional association between *B. variegata* L. and bees as frequent floral visitors. This pattern is consistent with that of flowering plants, which possess conspicuous petals, abundant nectar rewards, and exposed reproductive structures that facilitate pollen collection and transfer by bees [6,61,62]. Other scholars suggest that *B. variegata* L. exhibits a generalized pollination system but functional specialization through a strong reliance on honeybees, which serve as the principal pollinators and likely enhance pollen transfer, cross-pollination, and reproductive success [61,63]. Although pollination success depends not only on visitation rates but also on pollen-transfer efficiency, which was not studied in this research, the numerical dominance of honeybees suggests they likely make the greatest contribution to the reproductive success of *B. variegata* L. [6,61].

## 5. Discussion

### 5.1. Ecological Significance of Morphological Variation in Bauhinia variegata L. and Its Role in Floral Visitor Attraction

The variation in morphological characteristics observed among the sampled Bauhinia variegata trees reflects differences in developmental stage, resource allocation strategies, and ecological positioning within the stand. Structural attributes such as tree height, stem circumference, and canopy circumference are widely recognized as indicators of plant performance, competitive ability, and reproductive potential [40,41]. Trees with larger stem diameters and greater heights generally accumulate more biomass and possess a greater capacity to acquire and allocate resources to vegetative growth and reproduction [42,43]. For example, Tree 5 exhibited the largest stem circumference and one of the greatest heights, suggesting enhanced resource acquisition and growth. In contrast, Tree 8 had the largest canopy circumference, indicating a growth strategy that favours crown expansion and increased light interception. Such variation in canopy architecture has important ecological implications because larger canopies often support more extensive floral displays, which increase the visibility of flowering trees to floral visitors [44,46].

The observed relationships among stem size, canopy development, and tree height are consistent with established allometric principles governing tree growth and resource allocation [40,41,46]. However, deviations from these expected patterns suggest that local environmental conditions, competition, and individual growth strategies influence the distribution of resources between vegetative growth and reproductive investment [44]. These structural differences are likely to affect floral visitor attraction because floral abundance and display size are frequently associated with canopy development and plant vigour. Consequently, larger and structurally complex trees may support more flowers, provide greater resource availability, and attract a higher abundance of floral visitors.

The floral morphology of *B. variegata* further enhances its attractiveness to a diverse range of floral visitors. The species is characterized by large conspicuous petals, exposed reproductive structures, and accessible nectar resources that facilitate visitor access while promoting effective pollen transfer. Although moderate variation in petal dimensions was observed among sampled trees, the relatively stable lengths of stamens and styles suggest strong selective pressures maintaining reproductive functionality. Floral characteristics such as petal size, floral display, nectar accessibility, and the spatial arrangement of reproductive organs are well known to influence visitor preferences and visitation rates, thereby affecting plant reproductive success [6,47,62].

Honeybees recorded the highest mean abundance (102.1), whereas wasps, bee flies, and white-bellied sunbirds exhibited substantially lower mean abundances ranging between 11.8 and 12.8 individuals. Mormon butterflies were the least abundant floral visitors, with a mean abundance of 5.0 individuals. The significantly higher visitation frequency of honeybees suggests that the floral attributes of *B. variegata* are particularly suited to bee foraging behaviour.

The dominance of honeybees may be attributed to several floral characteristics of *B. variegata*, including its large floral display, exposed reproductive organs, and readily accessible nectar and pollen rewards. Honeybees possess specialized pollen-collecting structures, exhibit strong flower constancy, and frequently make repeated visits to rewarding floral resources. These behavioural and morphological adaptations increase contact with anthers and stigmas during foraging, making bees among the most effective and abundant floral visitors in many flowering plant systems [6,47,61]. Most significantly, honeybees are among the most important pollinators globally, contributing substantially to agricultural productivity and the reproduction of wild flowering plants [19,20].

It is worth noting that differences in visitor abundance may be explained by variation in floral reward preferences, body size, feeding mechanisms, and foraging strategies. White-bellied sunbirds, for example, primarily exploit nectar resources and may visit flowers less frequently than bees but can still facilitate pollen transfer owing to their larger body size and movement among flowers. Similarly, bee flies and butterflies may contribute to pollen movement while exploiting nectar resources, although potentially at lower frequencies than honeybees.

The interaction between floral morphology and visitor traits highlights the ecological importance of plant–visitor relationships in determining visitation patterns. Similar studies have demonstrated that floral traits such as flower size, colour display, nectar accessibility, and reproductive organ exposure interact with visitor morphology and foraging behaviour to influence visitation frequency and floral resource use [61,62,64].

All in all, the findings suggest that both structural and floral morphological traits of *B. variegata* play a significant role in shaping floral visitor assemblages and visitation abundance. Larger canopies and extensive floral displays likely enhance resource visibility, while floral characteristics such as exposed reproductive structures and accessible rewards promote frequent visitation.

### 5.2. Floral Rewards and Potential Pollination Roles of Visitors to Bauhinia variegata

It was apparent through the recorded results that honeybees (*Apis* spp.) were the most frequently observed floral visitors. However, this frequency of visitation alone does not necessarily imply pollinator specialization or coevolution [49]. It is worth noting that *Bauhinia variegata* flowers provide both pollen and nectar, supporting a diverse assemblage of floral visitors. Scholars such as Proctor et al. [65] and Willmer [66] argue that pollen benefits the pollinator with proteins, amino acids, lipids, and minerals that are essential for growth and reproduction. Moreover, nectar can provide carbohydrates that fuel flight and other energy-demanding activities. Consequently, floral visitors derive substantial nutritional benefits from exploiting these resources while simultaneously facilitating pollen transfer.

The production of nectar by *B. variegata* further suggests that visitors other than pollen-collecting honeybees may contribute to pollination. Nectar-feeding birds are recognised as effective pollinators in many flowering plant systems because they frequently contact floral reproductive structures during foraging and can transport pollen over relatively long distances, thereby promoting outcrossing and gene flow among plant populations [67,68]. Although bird visitation was not quantified in detail in the present study, their potential role as pollen vectors should not be overlooked, particularly given the conspicuous floral display and nectar rewards of *B. variegata*.

Furthermore, studies within the genus Bauhinia have demonstrated interactions with a broad spectrum of pollinators, including bees, butterflies, hummingbirds, bats, and other vertebrates, indicating that pollination systems within the genus are often generalized rather than highly specialized [22]. Therefore, while honeybees may represent important pollinators of B. variegata, the ecological significance of other floral visitors should also be recognized. Future studies examining visitor behaviour, pollen deposition, nectar utilization, and pollination efficiency would provide a more comprehensive understanding of the relative contributions of different floral visitors to the reproductive success of this species.

### 5.3. Novelty of the Study

The present study contributes novel insights into the reproductive ecology of *Bauhinia variegata* L. by addressing important knowledge gaps in plant floral–visitor interactions within southern Africa, a region that remains comparatively underrepresented in pollination ecology research. While numerous studies have examined plant–visitor interactions in Europe, North America, and Asia, empirical information from African urban and peri-urban landscapes remains limited [49,65,66,67]. By providing field-based data on floral morphology, floral visitor assemblages, and visitation abundance, this study expands the geographical scope of floral ecology research and contributes valuable baseline information for understanding species interactions and floral resource utilization in southern African ecosystems.

A further novel aspect of this study is the demonstration that *B. variegata* exhibits functional specialization despite possessing floral characteristics commonly associated with generalized pollination systems. This finding supports growing evidence that flowers exhibiting generalized morphological traits may nevertheless display ecological specialization through a disproportionate reliance on a subset of highly effective floral visitors [62,63]. The results, therefore, contribute to a growing body of literature demonstrating that visitation dominance and visitor effectiveness can be more influential than floral visitor richness alone in shaping plant reproductive outcomes [69,70].

The direct linkage established between floral morphological traits and floral visitor dominance represents another significant contribution of this study. The combination of large petals, conspicuous floral displays, accessible nectar rewards, and prominently exposed reproductive structures appears to favour honeybee visitation. Floral traits are increasingly recognized as ecological filters that influence visitor preferences, foraging behavior, resource exploitation, and visitation frequency, thereby shaping the structure of plant–visitor networks [6,61,62]. By integrating quantitative measurements of floral traits with records of floral visitation abundance, this study provides evidence that floral morphology plays an important role in determining floral visitor attraction and utilization patterns in *B. variegata*.

The study is also novel in its characterization of the floral visitor assemblage associated with *B. variegata* in southern Africa. The presence of other visitors, such as wasps, white-bellied sunbirds, bee flies, and Mormon butterflies, qualifies *B. variegata* as a supporter of the functionality of these visitors. This finding highlights the ecological importance of the species as a floral resource that attracts visitors with diverse feeding behaviours and foraging strategies. Similar studies have shown that diverse floral visitor assemblages can enhance the ecological stability of plant–visitor interactions and contribute to the persistence of pollination systems under changing environmental conditions [61,64].

Finally, this study provides one of the few quantitative assessments linking floral morphology, floral visitor assemblage composition, and visitation abundance in *B. variegata*. The integration of these components offers a more comprehensive understanding of how floral traits influence visitor recruitment and dominance patterns. By demonstrating that honeybees accounted for many floral visits despite the presence of multiple visitor groups, the study advances current understanding of visitor selection processes and reinforces the importance of floral morphology in mediating plant–visitor interactions [6,61,62]. Consequently, the findings establish an important foundation for future studies investigating floral visitor effectiveness, pollen transfer dynamics, fruit set, and reproductive success in *B. variegata* populations.

## 6. Conclusions

This study demonstrates that *Bauhinia variegata* serves as an important floral resource for a range of flower-visiting organisms. Honeybees were the most frequent visitors and showed the strongest association with the species. Nevertheless, the presence of wasps, white-bellied sunbirds, bee flies, and Mormon butterflies indicates that the tree supports a diverse assemblage of floral visitors. These findings highlight the ecological importance of *B. variegata* in providing floral resources that contribute to local biodiversity and justify further studies to evaluate the relative pollination effectiveness of the different visitor groups observed.

The findings further showed that floral morphology plays a critical role in shaping floral visitor attraction and visitation patterns. Large and conspicuous petals, accessible nectar resources, and prominently exposed reproductive structures appear to enhance floral attractiveness and favour frequent visitation by honeybees. In addition, variation in vegetative and floral traits among individual trees may influence visitor attraction by affecting floral display size, resource availability, and visibility.

## 7. Recommendations

While our study primarily focused on documenting floral visitors and their visitation frequencies, we acknowledge that birds may contribute significantly to pollination by foraging on floral nectar and unintentionally transferring pollen between flowers and trees. Indeed, the large, conspicuous flowers of *B. variegata* produce nectar that can attract nectar-feeding birds, and any contact with the reproductive structures during nectar collection may facilitate pollen transfer.

Future studies should move beyond visitation frequency and quantify floral visitor effectiveness by measuring pollen deposition, pollen transfer, fruit set, seed set, and reproductive success. Such investigations would enable a clearer assessment of the relative contributions of honeybees, white-bellied sunbirds, bee flies, wasps, and butterflies to the pollination biology of *B. variegata*. Additionally, further research should examine how variation in floral traits influences floral visitor preferences and reproductive outcomes across different environmental conditions and landscapes.

## Figures and Tables

**Table 1:** Diversity of pollinator species associated with Bauhinia variegata L. in the study area, categorized according to scientific name, common name, taxonomic order, and ecological function during floral visitation.

Photo	Scientific Name	Common Name	Order	Functional Role
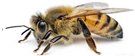 [50]	*Apis mellifera* L.	Western honeybee	Hymenoptera	Pollen collector
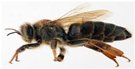 [51]	*Apis cerana* Fabricius	Asian honeybee	Hymenoptera	Pollen collector
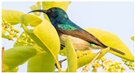 [51]	*Cinnyris talatala* Smith	White-bellied Sunbird	Passeriformes	Nectar/pollen feeder
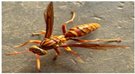 [Authors’ photograph]	*Polistes olivaceus* De Geer	Paper wasp	Hymenoptera	Nectar robber
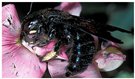 [Authors’ photograph]	*Xylocopa iris* Christ	Carpenter bee	Hymenoptera	Pollen collector
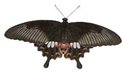 [Authors’ photograph]	*Papilio polytes* L.	Common Mormon butterfly	Lepidoptera	Pollen collector
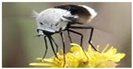 [Authors’ photograph]	*Bombomyia discoidea* Fabricius	Bee fly	Diptera	Nectar robber

## Data Availability

The data presented in this study are available on request from the corresponding author. The data are not publicly available due to ethical and confidentiality considerations.
